# An In Vivo Study on Brain Microstructure in Biological and Chronological Ageing

**DOI:** 10.1371/journal.pone.0120778

**Published:** 2015-03-25

**Authors:** Irmhild Altmann-Schneider, Anton J. M. de Craen, Annette A. van den Berg-Huysmans, Pieternella Slagboom, Rudi G.J. Westendorp, Mark A. van Buchem, Jeroen van der Grond

**Affiliations:** 1 Department of Radiology, Leiden University Medical Center, Leiden, The Netherlands; 2 Department of Gerontology and Geriatrics, Leiden University Medical Center, Leiden, The Netherlands; 3 Department of Molecular Epidemiology, Leiden University Medical Center, Leiden, The Netherlands; 4 Netherlands Consortium for Healthy Ageing, Leiden, The Netherlands; 5 Faculty of Health and Medical Sciences, Department of Public Health, University of Copenhagen, Copenhagen, Denmark; University G. D'Annunzio, ITALY

## Abstract

This study aimed to investigate whether magnetization transfer imaging (MTI) parameters of cortical gray and white matter and subcortical gray matter structures differ between subjects enriched for human familial longevity and control subjects to provide a thorough description of the brain phenotype of familial longevity. Moreover, we aimed to describe cerebral ageing effects on MTI parameters in an elderly cohort. All subjects were included from the Leiden Longevity Study and underwent 3 Tesla MTI of the brain. In total, 183 offspring of nonagenarian siblings, who are enriched for familial factors of longevity, were contrasted with 163 environmentally and age-matched controls. No differences in cortical and subcortical gray matter and white matter MTI parameters were found between offspring and control subjects using histogram-based and voxel-wise analyses. Cortical gray matter and white matter MTI parameters decreased with increasing chronological age (all p < 0.001). Decrease of white matter magnetization transfer ratio (MTR) was homogeneous throughout the whole mean white matter skeleton except for parts of the callosal splenium and partly the posterior limb of the internal capsule and superior region of the corona radiata (p < 0.05). Mean MTR of subcortical gray matter structures decreased with increasing age (p amygdala, caudate nucleus and putamen < 0.001; p pallidum = 0.001, p thalamus = 0.002). In conclusion, the brain phenotype of human familial longevity is - at a mean age of 66 years - not characterized by preserved macromolecular brain tissue integrity.

## Introduction

The human brain undergoes notable structural changes during the normal ageing process. The human lifespan is known to be influenced by genes as well as environmental factors [1] and it has been reported that extreme longevity is at least partly clustered in families [2]. Studying the brain phenotype in familial longevity is a valuable tool to learn more about the relevance of common age-related brain changes.

The Leiden Longevity Study was designed to investigate which factors in middle-aged humans associate with familial longevity. Offspring of long-lived nonagenarian siblings, who as a group are predisposed to become long-lived, are contrasted with their partners who represent the general population and serve as control subjects [3]. Compared to the control subjects, ageing in the offspring is marked by beneficial serum levels of lipid and thyroid parameters, preservation of insulin sensitivity, lower prevalence of myocardial infarction, hypertension, diabetes mellitus and use of cardiovascular medication and lower prevalence of cerebral white matter lesions (WMLs) and lacunar infarcts and preserved white matter microstructural integrity as measured using diffusion tensor imaging (DTI) [4–9]. Despite having the same chronological age, body mass index (BMI) and lifestyle [6] as the control subjects, the offspring generally display a younger biological age and for many other parameters appear healthier, such as for example better renal function [10] and a lower susceptibility to rheumatoid arthritis [11].

Recently, magnetization transfer imaging (MTI) has been put forward as a highly sensitive tool to study changes in the myelin content of the brain. MTI contrast is based on magnetization transfer between protons, which are bound to macromolecules and therefore restricted in motion, and protons in water, which can move freely [12]. Within the brain, myelin water contributes disproportionately to the magnetization transfer ratio (MTR), which therefore has been suggested to be a relatively specific measure of myelin integrity [13–15]. In recent studies it has been shown that MTR shows a steep decline in cortical and subcortical structures during lifespan (subjects aged 11 years-85 years) [16–24], indicating a decrease in myelin integrity. Still, in elderly subjects, 65 years of age and older, little MTI data are available [20–22]. Thus, the aim of this paper is twofold: To describe and partly reproduce cerebral ageing effects in MTI in an elderly cohort. Second to investigate whether the preserved white matter microstructural organization in human familial longevity is accompanied by preserved white matter tissue composition in the sense of preserved myelin integrity [25] to provide a thorough description of the brain phenotype of familial longevity.

## Materials and Methods

All subjects were included from the Leiden Longevity Study and underwent 3T MRI of the brain. The Leiden Longevity Study has been described more detailed elsewhere [3]. In short, 421 Dutch Caucasian families were enrolled in the study between 2002 and 2006 based on the following inclusion criteria: (1) there were at least two living siblings per family, who fulfilled the age criteria and were willing to participate, (2) men had to be aged ≥ 89 years and women had to be aged ≥ 91 years and (3) the sib pairs had to have the same parents. Additionally, offspring of these long-lived siblings were included as they have a 35% lower mortality rate compared to the general population. Their partners, who share the same socio-economic and geographical background, were enrolled as age-matched control group [3]. In the current study, 183 offspring of nonagenarian siblings, who are enriched for familial factors of longevity, were contrasted with 163 environmentally and age-matched controls.

The work described in this article has been carried out in accordance with the Code of Ethics of the World Medical Association (Declaration of Helsinki) and the study has been approved by the Medical Ethical Committee of the Leiden University Medical Center (Leiden, the Netherlands). Written informed consent was obtained from all subjects.

### MRI Acquisition

All imaging was performed on a whole body MR system with a field strength of 3T (Philips Medical Systems, Best, the Netherlands). The following images were acquired: 3DT1-weighted: TR = 9.7 ms, TE = 4.6 ms, FA = 8°, FOV = 224 x 177 x 168 mm, resulting in a nominal voxel size of 1.17 x 1.17 x 1.4 mm, covering the entire brain with no gap between slices.

T2-weighted: TR = 4200 ms, TE = 80 ms, FA = 90°, FOV = 224 x 180 x 144 mm, matrix size 448 x 320, 40 transverse slices to cover the entire brain with a slice thickness of 3.6 mm with no gap between slices.

FLAIR (fluid-attenuated inversion recovery): TR = 11000 ms, TE 125 ms, FA = 90°, FOV = 220 x 176 x 137 mm, matrix size 320 x 240, 25 transverse slices to cover the entire brain with a slice thickness of 5 mm with no gap between slices.

DTI (diffusion tensor imaging): TR = 9592 ms, TE = 56 ms, FA = 90°, FOV = 220 x 220 x 128 mm, matrix size 112 x 110, 64 transverse slices to cover the entire brain with a slice thickness of 2 mm with no gap between slices, 32 measurement directions, b-value = 1000.

MTI (magnetization transfer imaging): TR = 100 ms, TE = 11 ms, FA = 9°, FOV = 224 x 180 x 144 mm, matrix size 224 x 169, 20 transverse slices to cover the entire brain with a slice thickness of 7.2 mm with no gap between slices.

### Preprocessing and quantification of cortical gray matter, white matter and subcortical gray matter MTI parameters using histogram analysis

For analyses different tools of the Functional MRI of the Brain Software Library (FSL) software package were used [26, 27]. Raw magnetization transfer scans were split in the M0-sequence (acquired without saturation pulse) and the M1-sequence (acquired after application of a saturation pulse). 3D T1-weighted images were skull-stripped using BET (brain extraction tool) [28] and segmented using FAST (FMRIB’s automated segmentation tool) [29] and FIRST (FMRIB’s integrated registration and segmentation tool) [30], resulting in individual brain masks for white matter, cortical gray matter, amygdala, caudate nucleus, hippocampus, pallidum, putamen and thalamus. Individual 3D T1-weighted images were registered to the individual non-saturated M0 images using FMRIB’s linear image registration tool (FLIRT) [31, 32]. Registration matrices from the previous step were used to coregister the non-saturated M0 images and the individual brain masks for all above mentioned brain structures so that the MTR maps could be masked with these brain masks. To correct for possible partial volume effects, an eroded mask of these segmentations was created by removing one voxel in-plane for all mentioned volumes-of interest (VOIs) [33]. Individual MTR maps were calculated voxel by voxel following the equation MTR = (M0-M1)/M0 and MTR histograms were generated for all VOIs. Mean MTR, MTR peak height, normalized for the size of the VOI, and MTR peak location were calculated from each MTR histogram [34]. Mean MTR reflects the average MTR value per structure. MTR peak location reflects the most common MTR value. The peak height of the MTR histogram indicates the number of voxels showing the most common MTR value, and is a measure of uniformity of the underlying voxels in terms of MTR values. As the size of the VOI influences the number of voxels having the most common MTR value, MTR peak height was normalized by dividing the number of voxels with the most common MTR value by the number of voxels within the VOI. All MTI measures below -3 or above 3 standard deviations were excluded from statistical analysis.

### Automated segmentation of white matter lesions and quantification of MTR within these lesions

For the automated measurement of white matter lesion (WML) volume, 3DT1-weighted images were skull stripped [28] and the FLAIR and 3DT1 image were co-registered [31, 32]. The brain extracted FLAIR image was affine-registered to MNI152 standard space using FLIRT. A conservative MNI152 standard space white matter mask was used to extract the white matter from the FLAIR image. Subsequently, a threshold was set to identify which white matter voxels were hyperintense, followed by manually checking and editing for quality control. A mean MTR for all WML per subject was determined by masking the MTR maps with the registered WML masks.

### Voxel-based analysis of white matter MTR maps

Voxel-wise statistical analysis of white matter MTR data was performed using TBSS (Tract-Based Spatial Statistics) [35, 36], which was based on the creation of a mean white matter skeleton from fractional anisotropy (FA) images, derived from a DTI sequence. FA images were created by fitting a tensor model to the raw diffusion data using FDT (FMRIB’s diffusion tool box) and then brain-extracted using BET [28]. All subjects’ FA images were aligned into MNI152 standard space using non-linear registration (FNIRT) [37, 38]. A mean FA image (from the whole study population) was created and thinned to generate a mean FA skeleton which represents the centers of all tracts common to the group. The threshold for the mean FA skeleton was set to 0.2. All subjects’ non-saturated M0 images and saturated M1 images were linearly registered to the DTI B0 image, which is based on a raw T2 signal without diffusion weighting. MTR maps were calculated in “B0-space” in the same manner as described above. The resulting individual MTR maps were non-linearly registered into MNI152 standard space using FNIRT [37, 38] and projected onto the mean FA skeleton.

### Statistical Analysis

If not otherwise stated, data are presented as mean with standard deviation (SD) or mean with standard error (SE). Differences in sex, handedness, and history of hypertension, diabetes mellitus, myocardial infarction, stroke and malignancies between offspring and control subjects were calculated using Chi-square tests. Differences in age were tested using independent samples t-tests. MTI measures of all brain tissues were normally distributed. A linear regression model was used to assess the association of brain tissue MTI parameters with chronological age, adjusted for sex and affiliation to the offspring or control group. Analyses of differences in MTI parameters between offspring and control subjects were adjusted for age and sex and robust standard errors were calculated to correct for family relationships among the offspring. For statistical analyses, Statistical Package for the Social Sciences (SPSS) software for windows (version 20.0) was used. Robust standard errors were calculated with STATA software for windows (version 12; STATA).

For voxel-wise statistical analyses, the FSL randomise tool was used to perform permutation-based non-parametric testing (n = 5000 permutations) on the data. Threshold-Free Cluster Enhancement (TFCE) was applied to correct for spatial neighbourhood information, which is generally more robust and avoids the need for the arbitrary initial cluster-forming threshold. To correct for multiple comparisons, permutation based Family Wise Error (FWE) correction was used as a thresholding method. Significance was set at a TFCE FWE-corrected p < 0.05. Differences between offspring and control subjects were assessed, adjusted for age and sex. For the association between chronological age and MTI parameters, the same model was used though with contrasts of interest made for the age regressor instead of the two regressors describing group membership.

## Results

Characteristics of the study population are shown in Table 1. Imaging parameters were available for a total of 346 study participants, 183 offspring of long-lived siblings and 163 control subjects. The mean age of both groups was 66 years with a lower female percentage of 44% among offspring compared to 61% among control subjects (p = 0.002). More control subjects had a history of diabetes mellitus compared to offspring (7% versus 2%, p = 0.02). No differences were found concerning other morbidities.

**Table 1:** Characteristics of the study population.

	Offspring	Controls	P
	n = 183	n = 163	
**Demographics**			
Age (years), mean (SD)	66 (6.0)	66 (7.5)	0.82
Women, n (%)	99 (61)	81 (44)	**0.002**
Right-handedness, n (%)	162 (89)	146 (90)	0.76
**Comorbidities**			
Hypertension, n (%)	37 (20)	41 (25)	0.30
Diabetes mellitus, n (%)	4 (2)	12 (7)	**0.02**
Myocardial infarction, n (%)	3 (2)	4 (2)	0.60
Stroke, n (%)	4 (2)	3 (2)	0.85
Malignancies, n (%)	13 (7)	16 (10)	0.30

SD, standard deviation

### Brain tissue MTI parameters and biological age

Cortical and subcortical gray matter and white matter MTI parameters were quantified globally using histogram analysis. No differences were found between offspring and control subjects (Table 2). Also within white matter lesions MTI parameters were similar in offspring and control subjects: Mean MTR (SE): 35.26 (0.16) and 35.48 (0.17) respectively, p = 0.34; Mean normalized peak height (SE): 14.73 (5.39) and 14.82 (7.17) respectively, p = 0.85; Mean peak location: 37.33 (2.73) and 37.77 (1.99) respectively, p = 0.28. Moreover, the association of chronological age with brain tissue MTI parameters was not different between offspring and control subjects. Voxel-wise analysis of white matter MTR confirmed these results.

**Table 2:** MTI parameters of cortical and subcortical gray matter and white matter in offspring and control subjects.

	Mean (SE)		P
	Offspring	Controls	
	n = 183	n = 163	
**Gray matter**			
MTR (%)	33.51 (0.07)	33.50 (0.08)	0.61
Normalized peak height (%)	7.61 (0.09)	7.58 (0.08)	0.15
Peak location	36.23 (0.09)	36.40 (0.08)	0.14
**White matter**			
MTR (%)	39.41 (0.07)	39.43 (0.05)	0.97
Normalized peak height (%)	11.75 (0.17)	11.76 (0.17)	0.35
Peak location	40.62 (0.07)	40.73 (0.08)	0.23
**Amygdala**			
MTR (%)	36.72 (0.13)	36.98 (0.14)	0.92
Normalized peak height (%)	13.36 (0.21)	13.53 (0.22)	0.69
Peak location	38.15 (0.15)	38.48 (0.15)	0.22
**Caudate nucleus**			
MTR (%)	36.33 (0.14)	36.42 (0.17)	0.77
Normalized peak height (%)	8.96 (0.12)	8.91 (0.12)	0.32
Peak location	41.81 (0.14)	42.17 (0.20)	0.13
**Hippocampus**			
MTR (%)	38.36 (0.12)	38.45 (0.10)	0.73
Normalized peak height (%)	10.80 (0.16)	11.13 (0.17)	0.54
Peak location	40.72 (0.16)	40.96 (0.12)	0.20
**Pallidum**			
MTR (%)	38.37 (0.12)	38.36 (0.11)	0.61
Normalized peak height (%)	14.44 (0.23)	14.63 (0.23)	0.86
Peak location	38.72 (0.13)	38.75 (0.12)	0.46
**Putamen**			
MTR (%)	35.40 (0.12)	35.31 (0.12)	0.34
Normalized peak height (%)	12.82 (0.22)	12.99 (0.24)	0.81
Peak location	38.21 (0.11)	38.22 (0.11)	0.52
**Thalamus**			
MTR (%)	37.99 (0.14)	38.18 (0.18)	0.64
Normalized peak height (%)	10.93 (0.16)	11.06 (0.16)	0.87
Peak location	42.32 (0.13)	42.57 (0.18)	0.22

Values represent means (SE; standard error). P-values (p) are adjusted for age and sex and corrected for family relationships among the offspring.

MTR, magnetization transfer ratio

### Brain tissue MTI parameters and chronological age

MTI parameters were calculated globally for cortical gray matter, white matter, and subcortical gray matter structures using histogram analysis and their association with chronological age was assessed cross-sectionally. All cortical gray and white matter MTI parameters decreased with increasing age (p < 0.001) (Table 3). Mean MTR of all measured subcortical gray matter structures decreased with increasing age (p amygdala, caudate nucleus, pallidum and putamen < 0.001; p hippocampus = 0.02, p thalamus = 0.002). Mean normalized peak height decreased with increasing age in the amygdala, pallidum and putamen (p < 0.001). Mean peak location decreased in the pallidum and putamen (p = 0.03 and p = 0.001 respectively). Results are shown in Table 4. No differences in the association of chronological age with brain tissue MTI parameters were found between males and females except for mean MTR of the putamen, which showed a faster age-related decrease in men compared to women.

**Table 3:** Association of MTI parameters of cortical gray matter and white matter with chronological age.

	Standardized Beta	P
**Gray matter**		
MTR (%)	-0.37	**< 0.001**
Normalized peak height (%)	-0.24	**< 0.001**
Peak location	-0.20	**< 0.001**
**White matter**		
MTR (%)	-0.33	**< 0.001**
Normalized peak height (%)	-0.23	**< 0.001**
Peak location	-0.25	**< 0.001**

Values represent standardized Betas. P-values (p) are adjusted for sex and affiliation to the offspring or control group.

MTR, magnetization transfer ratio

**Table 4:** Association of MTI parameters of subcortical gray matter structures with chronological age.

	Standardized Beta	P
**Amygdala**		
MTR (%)	-0.23	**< 0.001**
Normalized peak height (%)	-0.18	**<0.001**
Peak location	-0.06	0.25
**Caudate nucleus**		
MTR (%)	-0.23	**< 0.001**
Normalized peak height (%)	-0.10	0.06
Peak location	-0.002	0.97
**Hippocampus**		
MTR (%)	-0.13	**0.02**
Normalized peak height (%)	-0.09	0.10
Peak location	-0.04	0.46
**Pallidum**		
MTR (%)	-0.19	**0.001**
Normalized peak height (%)	-0.21	**<0.001**
Peak location	-0.12	**0.03**
**Putamen**		
MTR (%)	-0.31	**< 0.001**
Normalized peak height (%)	-0.28	**< 0.001**
Peak location	-0.20	**< 0.001**
**Thalamus**		
MTR (%)	-0.17	**0.002**
Normalized peak height (%)	-0.06	0.27
Peak location	-0.04	0.51

Values represent standardized Betas. P-values (p) are adjusted for sex and affiliation to the offspring or control group.

MTR, magnetization transfer ratio

**Fig 1:**
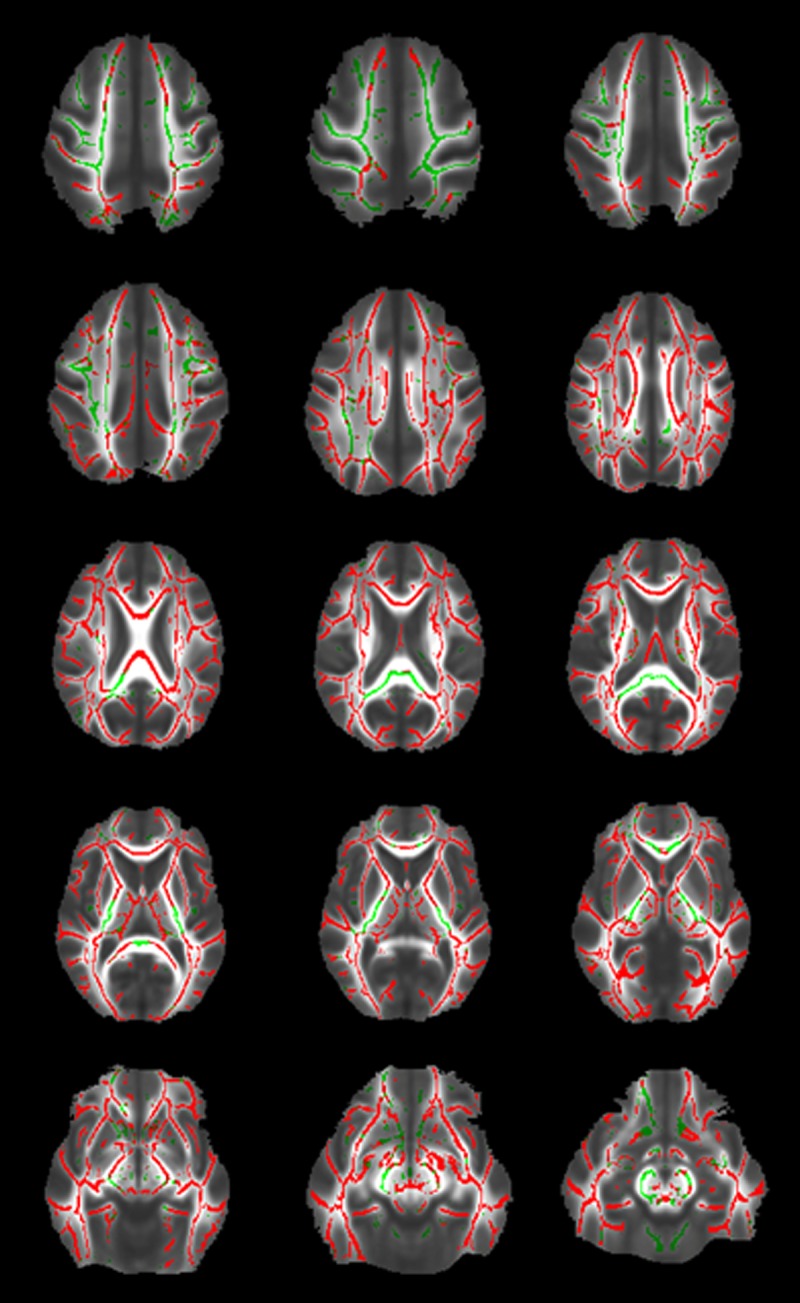
Voxel-based assessment of age-related changes of white matter magnetization transfer ratio (MTR). Fig. 1 shows results from the voxel-based assessment of age-related changes of white matter magnetization transfer ratio (MTR) in the whole study population using FSL-TBSS. Results are projected on the mean fractional anisotropy (FA) image of the whole study population which is derived from a diffusion tensor imaging (DTI) scan sequence. The mean white matter skeleton of the whole study population is shown in green color. Red color shows areas of statistically significant decrease of white matter MTR with increasing chronological age (p < 0.05).

FSL-TBSS was performed to assess the spatial distribution of age-related changes of white matter MTR (Fig. 1). White matter MTR decreased throughout the whole mean white matter skeleton except for partly the callosal splenium and posterior limb of the internal capsule and superior region of the corona radiata (p < 0.05).

## Discussion

The finding that no differences in any of the MTI parameters were found in any of the cortical and subcortical gray and white matter regions between partner and offspring is at least remarkable. Our findings indicate that preserved white matter integrity in familial longevity cannot be attributed to a lower susceptibility to white matter demyelination during the ageing process. Using DTI, we have shown recently that familial longevity is associated with preserved white matter integrity in the corpus callosum, as expressed in higher FA and lower radial diffusivity (RD) values in these regions [9]. This corresponds to a biological age benefit of 4.5 years [9]. The interpretation of the data of the current study is impossible without incorporating these previous findings.

During the past decade, brain ageing has been studied extensively using DT and MT imaging techniques and several attempts have been made to correlate *in vivo* MRI findings with knowledge from histopathological studies. FA, which reflects the extent of directionality of diffusion of water protons, is predominantly found to decrease with ageing and thought to be mainly influenced by axonal packing density as anisotropic diffusion also occurs in the absence of myelin [39]. With FA being relatively unspecific concerning underlying microstructural changes (e.g. axonal loss, axonal damage or demyelination) [40], several studies have included other DTI measures, such as RD and (axial diffusivity) AxD, to characterize the underlying processes. Studies in mice have suggested that RD might be a specific measure of myelin status [15, 41]. However, studies in humans have proven that increases in RD might not only be driven by loss of myelin but rather by a decrease in axon packing density through an increasing amount of extracellular water, which can be induced by different pathological processes [42]. Likewise, MTR has been proposed to be a relatively specific measure of white matter myelin status in humans [13], but has been shown to be additionally influenced by pathological conditions such as inflammation [43, 44], edema [44, 45] and axon loss [46]. With both RD and MTR solely being not specific to white matter myelin loss, the combination of increased RD and reduced MTR can be considered highly suggestive of reduced myelin content. In humans, different region-specific patterns of age-related changes in white matter DTI parameters have been described [42, 47]. In the corpus callosum which belongs to the predominantly frontally located late-myelinating regions consisting of thinner myelinated axons which are more prone to age-related myelin breakdown [48], an age-related decrease in FA was accompanied by increase in RD only or increase in RD as well as AxD [47, 49, 50]. This raises the possibility that both patterns reflect different degrees of severity of the same underlying structural changes [42]. MTR of the corpus callosum has been shown to decrease with increasing age [51]. Our previous data indicated that FA was decreased and RD increased in the callosal genu in offspring of nonagenarian siblings compared to control subjects, whereas AxD and MTR of the callosal genu were not different between groups. These findings might suggest that familial longevity is not associated with a lower susceptibility to white matter demyelination in the callosal genu and that the described difference between groups is caused by different susceptibility to underlying pathology other than demyelination. Against the background of the findings of Bennett et al. [42] however, another possible explanation could be that underlying changes are still too subtle to be depicted by MTR at this mean age [52].

Our findings, in which we specifically study ageing affects in an elderly cohort, add to the existing data in relatively younger subjects showing a steep decline in myelin integrity in cortical and subcortical structures during lifespan indicated by a steep MTR decrease with age [13, 19, 25].

One of the strengths of our study is the large study sample of middle-aged to elderly subjects. Secondly, the study design of comparing elderly individuals with a familial propensity to become long-lived to their partners is unique and allows us to gain more insight into which of the multiple age-related changes of the brain, frequently detected on MRI-scans in the general ageing population, are likely to be a relevant reflection of health. Thirdly, it should be noted that the partners of the offspring represent a control group matched for socio-economic and geographical background and lifestyle (as measured by FFQ).

The fact that study subjects were relatively young concerning age-related changes of the brain belongs to the limitations of this study. As differences between offspring and control subjects are likely to be small, a higher mean age of the study groups would probably facilitate the detection of possible differences.

In conclusion, this is the first study to compare macromolecular brain tissue integrity between elderly offspring of nonagenarian siblings and control subjects using MTI. Our findings suggest that, preserved white matter integrity of the callosal genu in familial longevity is not driven by a lower susceptibility to white matter demyelination during ageing. Moreover, at a mean age of 66 years, both offspring and control subjects show similar age-related decline of macromolecular brain tissue integrity throughout the whole brain suggesting that the phenotype of familial longevity at that age is not characterized by preserved macromolecular brain tissue integrity.
